# Experimental Parametric Model for Indirect Adhesion Wear Measurement in the Dry Turning of UNS A97075 (Al-Zn) Alloy

**DOI:** 10.3390/ma10020152

**Published:** 2017-02-10

**Authors:** Francisco Javier Trujillo, Lorenzo Sevilla, Mariano Marcos

**Affiliations:** 1 Department of Manufacturing Engineering, University of Malaga, C/ Dr. Ortiz Ramos s/n, Málaga 29071, Spain; lsevilla@uma.es; 2 Department of Mechanical Engineering and Industrial Design, University of Cadiz, Av. Universidad de Cádiz 10, Puerto Real 11519, Cádiz, Spain; mariano.marcos@uca.es

**Keywords:** aluminum alloys, UNS A97075, secondary adhesion wear, parametric models

## Abstract

In this work, the study of the influence of cutting parameters (cutting speed, feed, and depth of cut) on the tool wear used in in the dry turning of cylindrical bars of the UNS A97075 (Al-Zn) alloy, has been analyzed. In addition, a study of the physicochemical mechanisms of the secondary adhesion wear has been carried out. The behavior of this alloy, from the point of view of tool wear, has been compared to similar aeronautical aluminum alloys, such as the UNS A92024 (Al-Cu) alloy and UNS A97050 (Al-Zn) alloy. Furthermore, a first approach to the measurement of the 2D surface of the adhered material on the rake face of the tool has been conducted. Finally, a parametric model has been developed from the experimental results. This model allows predicting the intensity of the secondary adhesion wear as a function of the cutting parameters applied.

## 1. Introduction

In recent decades, the use of new materials—especially composite materials—has been gaining ground in the aircraft industry [1,2,3,4]. However, light alloys, such as aluminum alloys (mainly wrought alloys of 2000 and 7000 series) are still used in the manufacturing of structural parts of aircraft. On one hand, these alloys exhibit an excellent balance between physicochemical properties (mechanical strength, fracture toughness, fatigue performance, and corrosion resistance, among others) and weight [5,6]. On the other hand, the forming processes of these alloys show, in general, fewer problems than for composite materials [7,8,9,10]. In addition, most of the aeronautical projects for commercial aircraft usually have a lifetime about 20 years [11].

Consequently, these alloys are still used in the manufacturing of parts such as wing tension members, shear webs, and ribs. Specifically, the UNS A97075 (Al-Zn) alloy is extensively used in the upper skins and spar caps of wings, where the critical requirement is high compressive strength, whereas the requirements are not critical in tension loading or fatigue [12,13].

Most of these pieces are located in critical areas, so their probability of failure must be minimized. As a result, high quality requirements are demanded for these components, and their manufacturing must be carried out with narrow margins of deviation from design specifications [14].

The manufacturing of these parts usually involves different processes. Among them, machining is widely used [15,16]. This manufacturing process affects the surface integrity of machined parts (geometrical and physicochemical properties) which may result in a decrease of their functional performance [17,18]. In addition, the use of environmentally friendly technologies (such as dry machining) results in very tough conditions during the cutting process, both for the tool and the machined part, which may lead to significant deviations from the quality requirements [19,20,21].

Tool wear strongly influences the geometric deviations of machined parts, both at microscale (surface roughness) and macroscale (shape, size, and orientation) [22]. These deviations may limit the component quality or make the surface unacceptable. In this context, tool wear becomes an especially important factor, which affects not only the functional performance of machined parts but also the economic and energetic performance of the process [23,24].

For all the above, it is necessary to know the physicochemical mechanisms that involves the tool wear process, and also what factors most influences its intensity. In this regard, the indirect adhesion wear is the dominant wear mechanism when aeronautical aluminum alloys are machined [25,26]. This wear mechanism is caused by the incorporation of fragments of the workpiece material to the tool, both in the cutting edge (Built-Up-Edge, BUE), as on the rake face (Built-Up-Layer, BUL), which causes geometrical and physicochemical alterations of the tool. Furthermore, a loss of tool material and an abrasion process on the tool rake face occur when this material is removed.

This indirect adhesive wear mechanism has been extensively studied for UNS A92024 (Al-Cu) alloy and UNS A97050 (Al-Zn) alloy, and slight differences have been found in the way that BUL and BUE is generated [27,28,29]. However, this mechanism has been less studied for UNS A97075 (Al-Zn) alloy. On the other hand, several studies which analyze the influence of cutting speed and feed on the intensity of tool wear of these alloys have been found in the literature [30,31]. Notwithstanding, there is a lack of research dealing with the study of the influence of the depth of cut. In addition, most of these studies were conducted in qualitative terms. Thus, no studies have been found devoted to obtaining parametric models to quantify the intensity of wear as a function of the cutting parameters (cutting speed, feed, and depth of cut).

In this work, the influence of cutting parameters (cutting speed, feed, and depth of cut) on the tool wear is studied in the dry turning of cylindrical bars of the UNS A97075 (Al-Zn) alloy. In addition, the secondary adhesion wear mechanism of this alloy is checked, and its behavior is compared with similar, more studied alloys. Finally, a parametric model is formulated which allows to predict the secondary adhesion wear intensity as a function of cutting parameters. It should be pointed that this model is a first approach to the measurement of the 2D area of the adhered material on the rake face of the tool. A more accurate calculation (3D) is postponed for further research.

## 2. Materials and Methods

Several machining test were carried out using cylindrical bars (*L* = 200 mm and *D* = 30–60 mm) of UNS A97075 (Al-Zn) Alloy. Table 1 shows the mass percentage of the main elements contained in the tested alloy. This composition was obtained using arc AES (Atomic Emission Spectroscopy) techniques.

**Figure 1:**
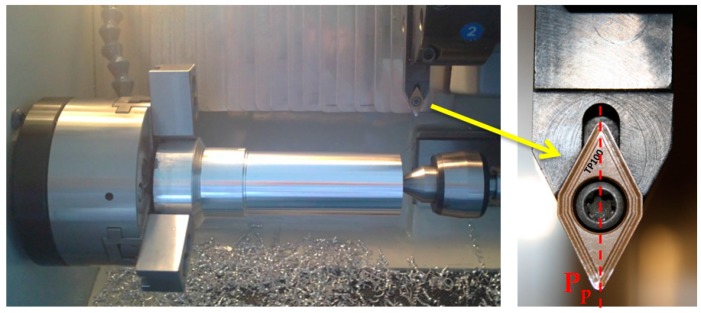
Turning test setup.

The machining operation selected was horizontal turning, in order to minimize the influence of the geometrical features of the process. The tests were conducted in a CNC turning center, Figure 1. Taking into account the use of environmentally friendly techniques, all tests were performed dry.

**Figure 2:**
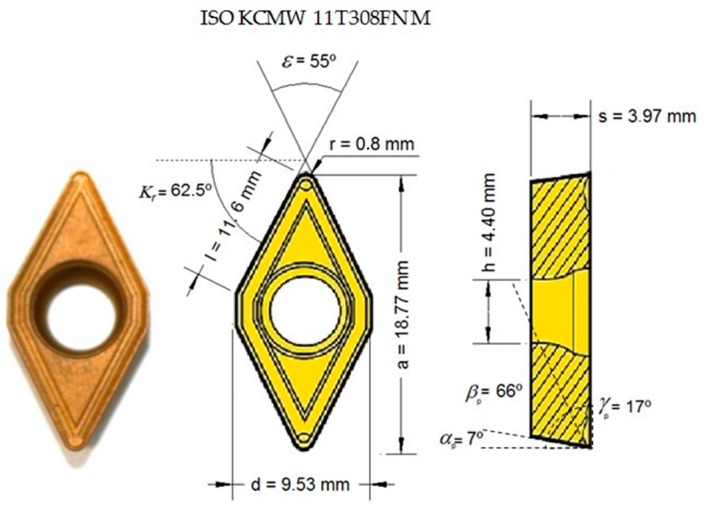
Tool geometry.

The tools used were WC inserts coated with TiN (ISO KCMW 11T308FN M). Tool geometry is shown in Figure 2 and the cutting angle configuration used is shown in Table 2. All tests were carried out using a new tool, with the aim of ensuring the same initial conditions.

Different combinations of cutting parameters values (cutting speed (*v*_c_), feed (*f*), and depth of cut (*a*_p_)) were used in order to analyze their influence on tool wear. The values applied are shown in Table 3. These values are commonly used in machining of these alloys for particular aeronautical applications [14,31]. In this context, it must be pointed that, although low cutting speeds are not recommended for machining aluminum alloys, these alloys are often hybridized with other materials in which these low cutting speeds are required. Aiming to analyze the initial tool changes caused by secondary adhesion wear, all tests lasted *t* = 10 s.

**Figure 3:**
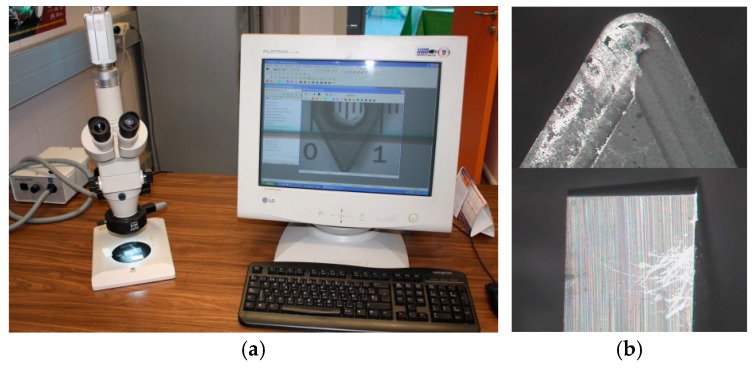
(**a**) SOM equipment setup; (**b**) Rake and flank face SOM images.

The cutting process was monitored online by using a digital camera. Furthermore, the obtained chip was collected, photographed, and stored for later analysis. On the other hand, changes in the rake and flank insert faces were monitored offline by using several microscopy techniques. First of all, tools were prepared for observation by Stereoscopic Optical Microscopy (SOM) using a stereoscopic microscope (63×), image base camera, and capture card, Figure 3. The captured tool images were analyzed using image processing software. Further analysis in the micro-compositional and micro-structural tool changes were performed by combining Scanning Electron Microscopy (SEM) and Energy Dispersive Spectroscopy (EDS) techniques, using a scanning electron microscope attached to an energy dispersive spectrometer analyzer. Finally, a first approach to the measurement of the surface of material adhered on the tool rake face was carried out using image processing software.

## 3. Results and Discussion

**Figure 4:**
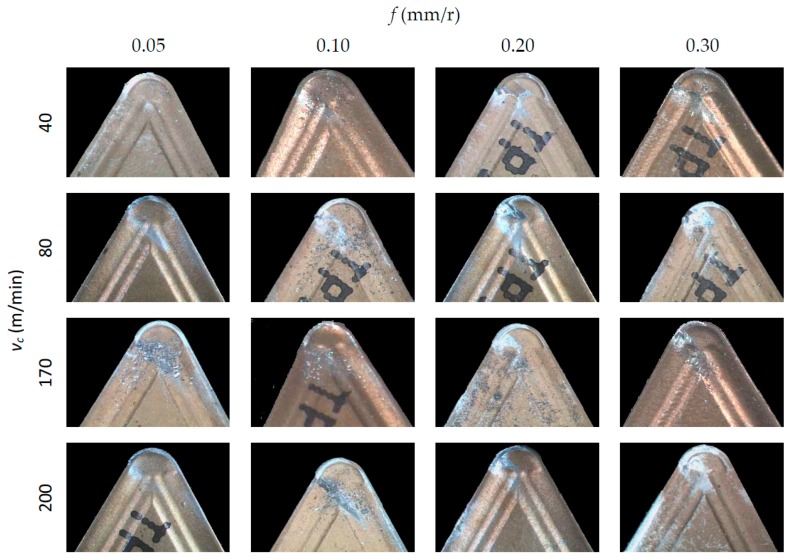
SOM images (30×) of the rake face, for *a*_p_ = 0.5 mm.

**Figure 5:**
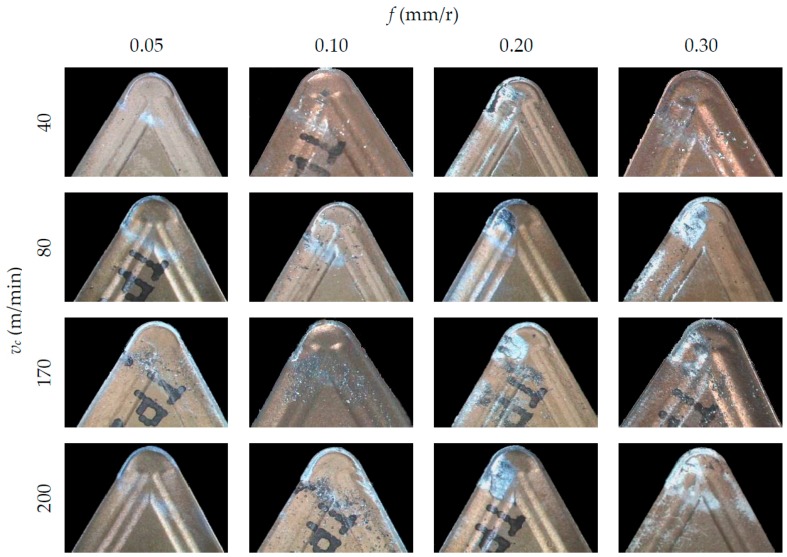
SOM images (30×) of the rake face, for *a*_p_ = 1 mm.

**Figure 6:**
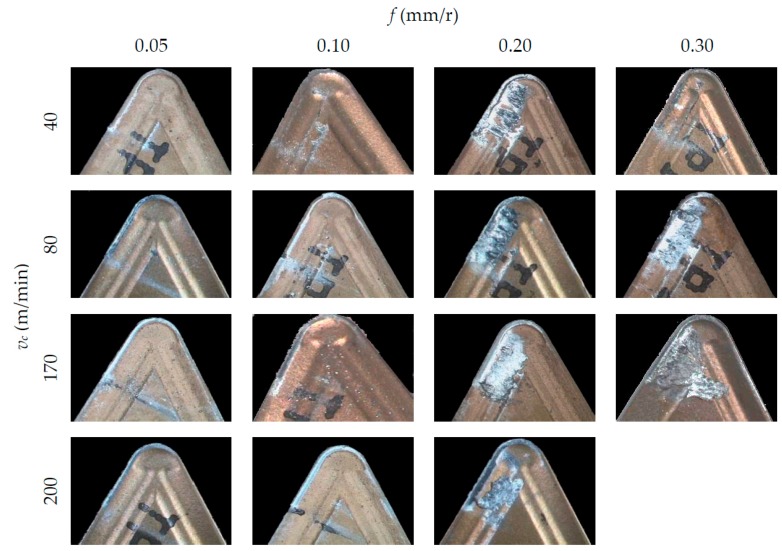
SOM images (30×) of the rake face, for *a*_p_ = 2 mm.

Figure 4, Figure 5 and Figure 6 show SOM images from the rake face of the tools used in the machining tests, as a function of *v*_c_ and *f*, for each *a*_p_ applied.

In general, an incorporation of the material from the workpiece to the tool can be observed, both on the rake face (BUL, Built-Up-Layer) and in the edge of the tool (BUE, Built-Up-Edge). These incorporations of material cause an alteration in the tool initial geometry and its physicochemical properties, giving rise to an indirect adhesion wear [32]. It is apparent, from these figures, that BUL and BUE intensity are higher in the highest range of *f* applied (0.20 and 0.30 mm/r), regardless of *v*_c_. In this sense, no substantial changes are noted when *f* values increase from 0.05 up to 0.10 mm/r. This behavior is similar for all the values of *a*_p_ tested. In terms of *v*_c_, no significant changes are observed in the quantity of material adhered to the tool when *v*_c_ is modified. Only for *f* = 0.30 mm/r can an increment in the BUL intensity be observed when *v*_c_ is increased from 40 m/min up to 80 m/min. This trend is similar for all values of *a*_p_ used. Regarding of the influence of *a*_p_, the intensity of the indirect adhesion wear shows a trend to increase when *a*_p_ increases, mainly in the range of higher values of *f* (0.20 and 0.30 mm/r). Nevertheless, its influence is less noticeable than the influence of *f*.

For all the above mentioned, it can be said that the feed is the parameter that most influences the intensity of the adhesion effects, followed by the depth of cut. This can be explained taking into consideration that an increasing of *f* and *a*_p_ values results in higher values of cutting forces and compression stresses in the cutting area. As a result, an increase in temperature occurs in the cutting edge, enabling the melting of the work material and its adhesion to the tool due to thermomechanical effects. On the other hand, the cutting speed shows a lower influence on the adhesion effects because of its lesser influence in the cutting forces values.

**Figure 7:**
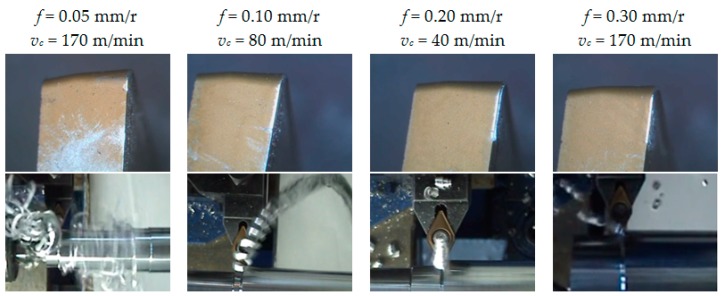
SOM images (20×) of the flank face and captured images of the chip, for *a*_p_ = 2 mm.

Figure 7 collects images from the flank face of the tools and the chip generated in the cutting process, for some of the tests performed. As it can be observed, the flank face shows an incipient stage of abrasion wear for the lower values of *f* (0.05 and 0.10 mm/r), whereas this abrasion effect is less noticeable for the higher values of *f* (0.20 and 0.30 mm/r).

This fact can be explained by taking into consideration the morphology of the chip generated in the cutting process. The chip obtained for higher feed values is shorter than for lower feed values, where it appears longer and more flexible, with a clear trend to form chip nests. This longer chip strikes on the flank face of the tool, resulting in the aforementioned abrasion wear. These observations were similar for all *a*_p_ tested values, regardless of *v*_c_.

It should be noted that the observed behavior in this alloy is in good agreement with the results obtained by other researchers on similar alloys, such as UNS A92024 (Al-Cu) and UNS A07050 (Al-Zn) alloys, both of them widely used for aeronautical applications [31]. Notwithstanding, slight differences between them can be appreciated. No significant changes are observed in the amount of adhered material on the rake face when *v*_c_ is modified, for the UNS A97050 and UNS A97075 alloys, whereas these changes are more noticeable for the UNS A92024 alloy, mainly for the lowest values of *f* (0.05–0.10 mm/r).

BUE is usually unstable, detaching and reappearing cyclically, often due to the action of the chip. This fact is more likely when the chip is longer and more flexible, as it happens when low values of *f* are applied for machining UNS A97050 and UNS A97075 alloys. However, UNS A92024 alloy is more fragile, due to its higher mass percentage of Cu and lower mass percentage of Zn. As a result, the chip obtained is more segmented and shorter, so that the BUE instability is lower. For this last alloy, BUE becomes larger and its mechanical instability increases when *v*_c_ increases. Because of this, the influence of *v*_c_ on indirect adhesion wear is slightly higher for UNS A92024 alloy.

On the other hand, there are some differences in the secondary adhesion wear mechanisms between UNS A92024 and UNS A97050 alloys. In both alloys, a primary BUL appears in the early moments of machining due to thermomechanical causes. This first layer, with a composition close to the pure aluminum, causes alterations in the geometry and physicochemical properties of the cutting tool. As a result, a first BUE, with a composition close to the alloy, appears due to mechanical causes. This BUE grows to a critical thickness and is extruded to result in a secondary BUL. In the case of UNS A92024 alloy, this secondary BUL has a composition close to the pure aluminum and arises due to thermomechanical causes. In contrast, for UNS A97050 alloy, the secondary BUL exhibits a very similar composition to the alloy and is originated by mechanical causes. In addition, the BUE size is smaller for UNS A97050 alloy, due to its higher plasticity. Finally, this cycle is repeated in both alloys, resulting in successive overlapping layers of secondary BUL [16,26,27,28].

**Figure 8:**
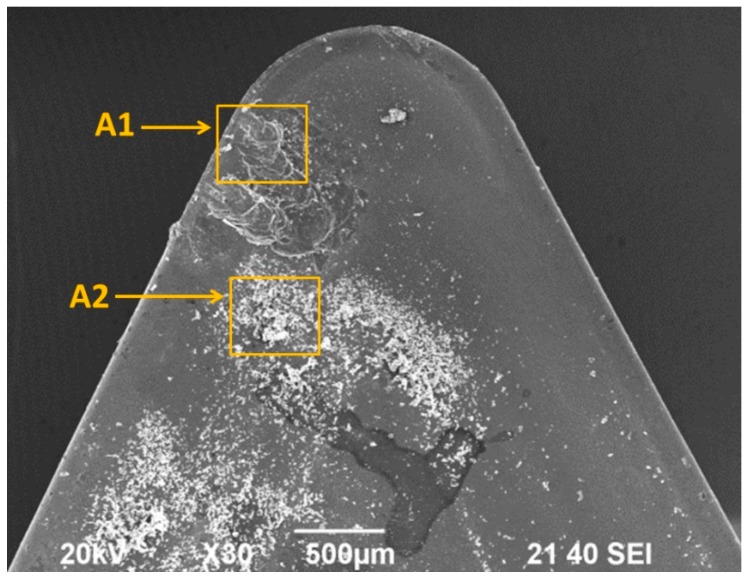
SEM image from the rake face (*v*_c_ = 170 m/min, *f* = 0.20 mm/r and *a*_p_ = 1 mm).

In order to check whether the behavior of UNS A97075 alloy is similar to UNS A97050 alloy, various SEM/EDS analysis were performed. Figure 8 shows a SEM image from the rake face of one of the tools used in the tests. Two different areas have been analyzed: An area (A1) close to the cutting edge, where a stratified BUL can be observed, and another one (A2), further inside the rake face, where small material particles can be observed.

**Figure 9:**
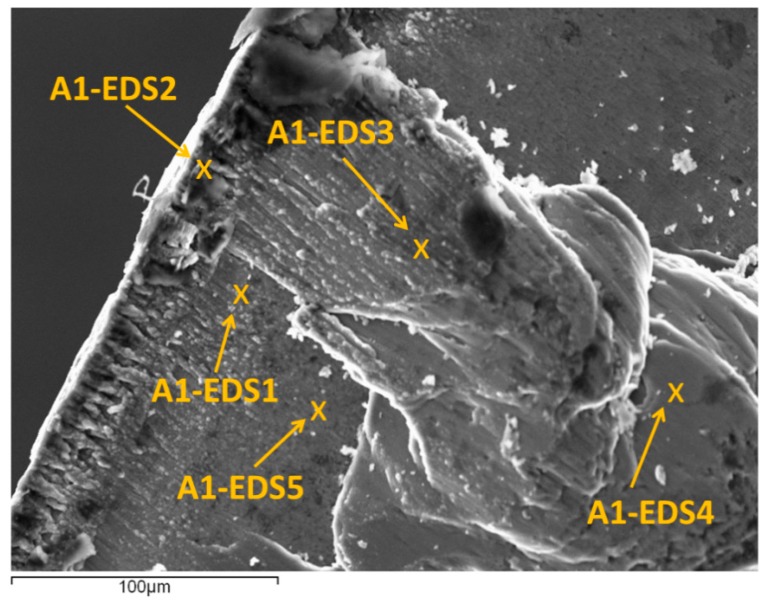
SEM image from A1 (the analyzed points are marked).

Figure 9 shows a SEM image from A1, where the different points analyzed has been marked. These points have been selected in areas where adhesion phenomena can be observed (close to the cutting edge and on the rake face), as well as in areas where adhesion is not present. Table 4 presents a comparison between the alloy composition (mass %) and those obtained from each EDS analysis.

The spectrum A1-EDS1 corresponds to a thin layer of material on the rake face, close to the cutting edge. A decrease of intermetallic (Zn, Mg, and Cu) regarding the UNS A97075 Alloy can be observed, as well as an increasing of Al, with a composition close to pure aluminum in this point (Table 4). Because of this, it can be said that this point is on the primary BUL, which appears due to thermomechanical causes.

The spectrum A1-EDS2, located in the cutting edge, shows a percentage of intermetallic (Zn, Cu, and Mg) very similar to UNS A97075 alloy (Table 4). This point corresponds to BUE, which appears mainly due to mechanical causes.

The spectrums A1-EDS3 and A1-EDS4 are located on the rake face, on overlapped layers of adhered material, farther from the cutting edge. These points correspond to the secondary BUL, which is obtained by the extrusion of the BUE. The composition in these points is close to UNS A97075 alloy (Table 4), which suggests that secondary BUL appears only due to mechanical causes. Finally, the spectrum A1-EDS5 is located in a point where secondary adhesion wear is not present, and its composition is close to the coating tool, TiN (Table 4).

**Figure 10:**
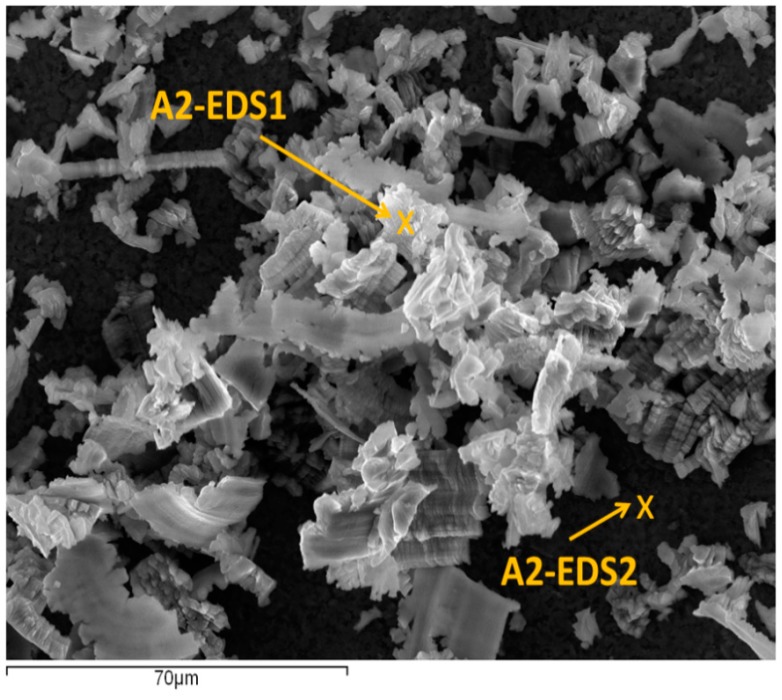
SEM image from A2 (the analyzed points are marked).

Figure 10 shows an SEM image from A2, where two different points have been marked. Table 5 shows a comparison between the alloy composition (mass %) and the EDS analysis in both points.

The spectrum A2-EDS1 (Table 5) shows a composition very similar to the UNS A97075 alloy. It corresponds to small particles of alloy which have been detached from the chip and have impacted on the rake face of the tool. As a result, an incipient abrasion wear can be observed. The spectrum A2-EDS2 shows a composition close to the coating of the tool, TiN (Table 5).

It should be pointed out that all these observations were similar for the different values of cutting parameters that have been considered. For all the above, it can be said that the secondary adhesion wear mechanism is similar in the UNS A97075 and UNS A97050 alloys.

**Figure 11:**
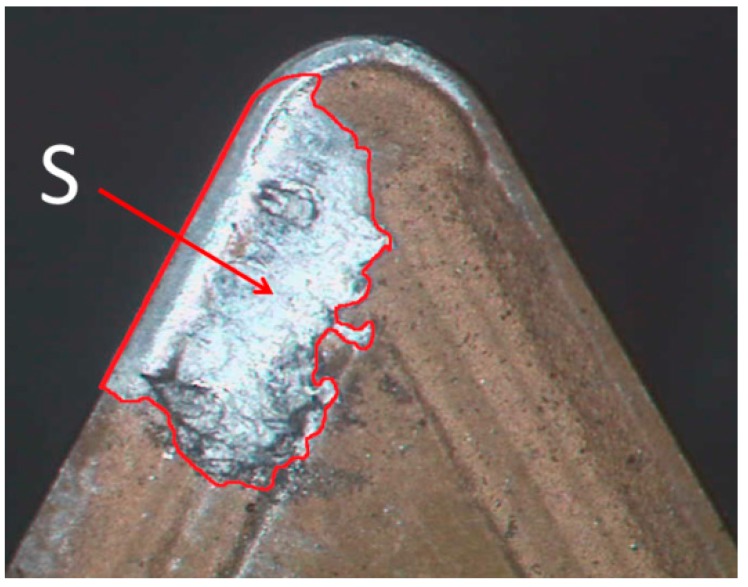
SOM image (30×) of the measurement process of *S (f* = 0.20 mm/r, *v*_c_ = 170 m/min and *a*_p_ = 2 mm).

Once the secondary adhesion wear mechanism has been analyzed, the amount of material adhered to the rake face was measured, in order to study the influence of the cutting parameters on the tool wear. To do this, in a first approach, the 2D surface (*S*) occupied by the BUL was measured (an example is shown in Figure 11).

**Figure 12:**
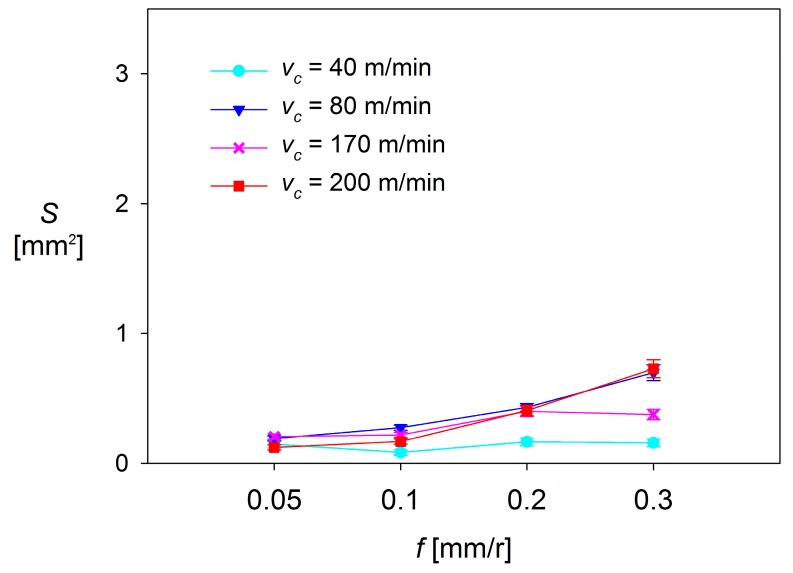
*S* = f(*v*_c_, *f*), for *a*_p_ = 0.5 mm.

**Figure 13:**
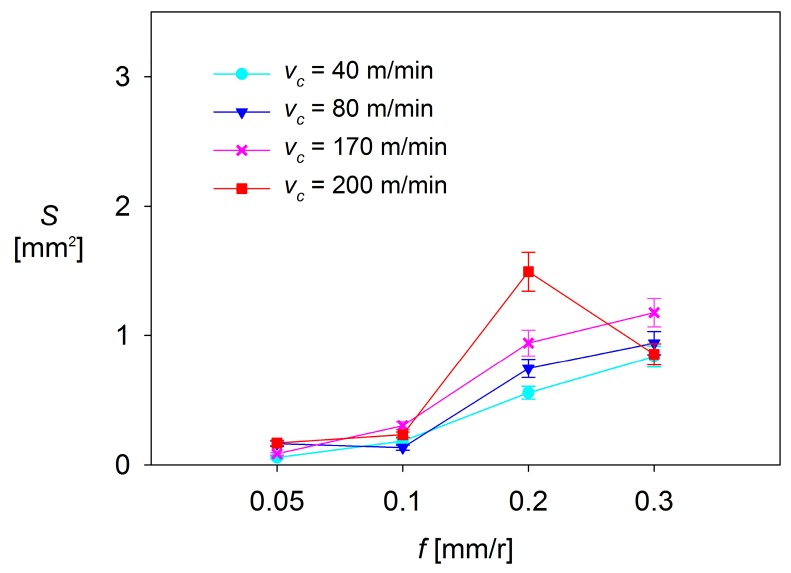
*S* = f(*v*_c_, *f*), for *a*_p_ = 1 mm.

**Figure 14:**
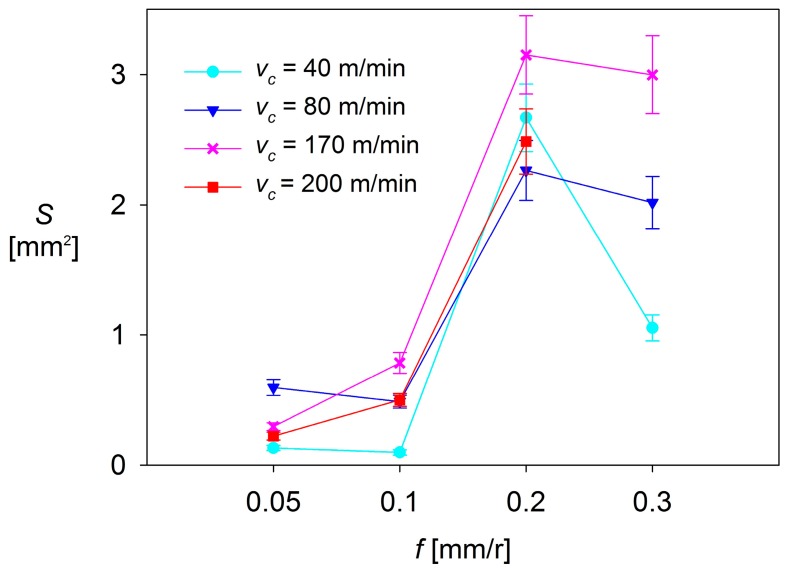
*S* = f(*v*_c_, *f*), for *a*_p_ = 2 mm.

Figure 12, Figure 13 and Figure 14 plot *S* as a function of *f* and *v*_c_, for each *a*_p_ tested.

As it can be observed, there are no significant changes in *S* when low values of *f* are applied (0.05–0.10 mm/r). For *a*_p_ = 0.5 mm and *a*_p_ = 1 mm, *S* shows a general trend to remain constant, regardless of *f* and *v*_c_. These changes are slightly higher for *a*_p_ = 2 mm, where *S* shows a trend to increase when *f* increases from 0.05 mm/r up to 0.10 mm/r, in the range of the highest values of *v*_c_ used (170 and 200 m/min). In contrast, *S* tends to decrease with *f* when lower values of *v*_c_ are applied (40 and 80 m/min).

On the other hand, the values of *S* observed in the highest range of *f* (0.20–0.30 mm/r) are much larger than those observed in the lowest range of *f* (up to 5 times higher for *a*_p_ = 0.5 mm, 10 times for *a*_p_ = 1 mm, and 15 times for *a*_p_ = 2 mm), regardless of *v*_c_. For *a*_p_ = 0.5 mm and *a*_p_ = 1 mm, the values of *S* show a general trend to remain constant or to slightly increase with *v*_c_. Only a decrease of *S* is observed for *v*_c_ = 200 m/min, when *f* is increased from 0.20 mm/r up to 0.30 mm/r. Something similar occurs for *a*_p_ = 2 mm, where *S* shows a general trend to decrease, regardless of *v*_c_. This fact can be explained by taking into consideration that the combination of high values of *v*_c_, *f* and *a*_p_ results in a high instability of the adhered material, which tends to become detached.

Because of all previously observations, it can be said that the cutting parameter that most influences *S* is *f*, resulting in a notable increase of *S* when going from low range values of *f* (0.05 and 0.10 mm/r) to high range values (0.20 and 0.30 mm/r). This behavior is similar to that shown by other process output variables, discussed in previous research [14,31,32], such as surface quality (measured in terms of arithmetic average roughness, *Ra*). The second most influential parameter is *a*_p_, which enhances the effect of *f* when its value increases. Finally, the less influential parameter is *v*_c_, which only shows a limited relevance when it is combined with high values of *f* and *a*_p_.

The experimental results suggest that it is possible to obtain a parametric model of *S* = f(*v*_c_, *f*, *a*_p_). For this purpose, different models (potential, exponential and polynomial) were tested. The best fit was obtained for a potential model as follows (Equation (1)):

(1) *S* = *K*∙*v_c_^x^*∙*f^y^*∙*a*_p_^*z*^,

Coefficient *K* and exponents *x*, *y*, and *z* were calculated through a linear regression of experimental data, obtaining a model as follows (Equation (2)):

(2) *S* = 0.592∙*v*_c_^0.404^∙*f*^1.085^∙*a*_p_^0.885^,

The results obtained for the values of the exponents are in good agreement with all the above exposed. On the one hand, *S* exhibits a higher dependence on *f*, due to the higher value of the exponent *y*. On the other hand, *z* shows a lower value than *y*, thus *S* exhibits a lower dependence on *a*_p_. Finally, the lowest value is obtained for *x*, so *v*_c_ has the lowest influence on *S*. It should be highlighted that this model has shown an acceptable fit to the experimental results (*R* squared close to 0.8). However, this is a first approach to the measurement of the secondary adhesion wear. A more accurate calculation (3D) is postponed for further research.

## 4. Conclusions

In this work, the influence of cutting parameters (cutting speed, feed, and depth of cut) on the tool wear in the dry turning of cylindrical bars of the UNS A97075 (Al-Zn) alloy is analyzed. The experimental results have shown that indirect adhesion wear is the predominant wear mechanism. In most tests conducted, it can be seen that work material adhered both in the cutting edge (BUE) and on the rake face of the tool (BUL).

It has been found that the feed is the parameter that most influences the intensity of the adhesion effects, followed by the depth of cut. On the other hand, the cutting speed has shown a lower influence on the adhesion effects because of its lesser influence in the cutting force values.

Furthermore, the flank face of the tool has shown an incipient stage of abrasion wear for the lower values of feed applied, regardless of cutting speed and depth of cut. For lower feed values, the chip appears longer and flexible, with a tendency to form chip nests which strike on the flank face of the tool, resulting in abrasion wear.

It should be noted that the observed behavior in this alloy is in good agreement with the results obtained by other researchers on similar alloys, such as UNS A92024 (Al-Cu) and UNS A07050 (Al-Zn) alloys. Notwithstanding, this research presents the novelty of analyzing the influence of the depth of cut. Thereby, the experimental results have revealed that the depth of cut enhances the effect of feed when its value increases.

A study of the physicochemical mechanisms of the secondary adhesion wear is carried out. The behavior of this alloy is compared to similar aeronautical aluminum alloys, such as the UNS A92024 (Al-Cu) alloy and UNS A97050 (Al-Zn) alloy. It has been shown that the secondary adhesion wear mechanism is similar in the UNS A97075 and the UNS A97050 alloys and slightly different for the UNS A92024 alloy.

A first approach to the quantifying of the secondary adhesion wear intensity is performed. For this purpose, the 2D surface of the adhered material on the rake face of the tool has been measured. As a result, an experimental parametric model has been developed. This model allows prediction of the intensity of the secondary adhesion wear as a function of the cutting parameters applied.

## Figures and Tables

**Table 1:** Composition of machined alloy (mass %).

Zn	Mg	Cu	Cr	Si	Mn	Al
6.03	2.62	1.87	0.19	0.09	0.07	Rest

**Table 2:** Cutting angles setup (°).

Flank angle (α_p_)	7
Wedge angle (β_p_)	66
Rake angle (γ_p_)	17
Tool cutting edge angle (κ_r_)	62.5
Insert included angle (ε)	55

α_p_, β_p_ and γ_p_ are defined in the tool back plane (*P*_p_, Figure 1).

**Table 3:** Cutting parameters values used in the turning tests.

***v_c_*** [m/min]	40	80	170	200
***f*** [mm/r]	0.05	0.10	0.20	0.30
***a_p_*** [mm]	0.5	1	2	-

**Table 4:** Composition from EDS analysis (A1).

EDS Analysis	Composition	C	N	O	Mg	Al	Ti	Cu	Zn
**Alloy tested UNS A97075**	Mass [%]	-	-	-	2.62	88.92	-	1.87	6.03
	(Mass/Al) [%]	-	-	-	2.94	100	-	2.10	6.78
**A1-EDS1**	Mass [%]	18.31	-	4.01	0.62	58.09	17.99	-	0.98
	(Mass/Al) [%]	-	-	-	1.06	100	-	-	1.69
**A1-EDS2**	Mass [%]	9.23	-	1.72	2.40	79.81	-	1.30	5.54
	(Mass/Al) [%]	-	-	-	3.00	100	-	1.62	6.94
**A1-EDS3**	Mass [%]	5.12	-	1.61	2.51	83.61	-	1.49	5.66
	(Mass/Al) [%]	-	-		3.00	100	-	1.78	6.76
**A1-EDS4**	Mass [%]	1.35	-	0.95	2.61	87.49	-	1.51	6.09
	(Mass/Al) [%]	-	-	-	2.98	100	-	1.72	6.96
**A1-EDS5**	Mass [%]	-	21.8	-	-	14.31	63.89	-	-
	(Mass/Al) [%]	-	-	-	-	-	-	-	-

**Table 5:** Composition from EDS analysis (A2).

EDS Analysis	Composition	C	N	O	Mg	Al	Ti	Cu	Zn
**Alloy tested UNS A97075**	Mass [%]	-	-	-	2.62	88.92	-	1.87	6.03
	(Mass/Al) [%]	-	-	-	2.94	100	-	2.10	6.78
**A2-EDS1**	Mass [%]	4.56	1.10	3.02	2.51	80.88	-	2.01	5.92
	(Mass/Al) [%]	-	-	-	3.10	100	-	2.48	7.30
**A2-EDS2**	Mass [%]	11.80	22.80	1.11	-	2.91	61.38	-	-
	(Mass/Al) [%]	-	-	-	-	-	-	-	-
